# Capillary recruitment in a theoretical model for blood flow regulation in heterogeneous microvessel networks

**DOI:** 10.1002/phy2.50

**Published:** 2013-08-22

**Authors:** Brendan C Fry, Tuhin K Roy, Timothy W Secomb

**Affiliations:** 1 Program in Applied Mathematics, University of ArizonaTucson, Arizona, 85721; 2 Department of Anesthesiology, Mayo ClinicRochester, Minnesota, 55905; 3 Department of Physiology, University of ArizonaTucson, Arizona, 85724

**Keywords:** Blood flow, capillary, computer modeling

## Abstract

In striated muscle, the number of capillaries containing moving red blood cells increases with increasing metabolic demand. This phenomenon, termed capillary recruitment, has long been recognized, but its mechanism has been unclear. Here, a theoretical model for metabolic blood flow regulation in a heterogeneous network is used to test the hypothesis that capillary recruitment occurs as a result of active control of arteriolar diameters, combined with unequal partition of hematocrit at diverging microvascular bifurcations. The network structure is derived from published observations of hamster cremaster muscle in control and dilated states. The model for modulation of arteriolar diameters includes length-tension characteristics of vascular smooth muscle and responses of smooth muscle tone to myogenic, shear-dependent, and metabolic stimuli. Blood flow is simulated including nonuniform hematocrit distribution. Convective and diffusive oxygen transport in the network is simulated. Oxygen-dependent metabolic signals are assumed to be conducted upstream from distal vessels to arterioles. With increasing oxygen demand, arterioles dilate, blood flow increases, and the numbers of flowing arterioles and capillaries, as defined by red blood cell flux above a small threshold value, increase. Unequal hematocrit partition at diverging bifurcations contributes to recruitment and enhances tissue oxygenation. The results imply that capillary recruitment, as observed in the hamster cremaster preparations, can occur as a consequence of local control of arteriolar tone and the resulting nonuniform changes in red blood cell fluxes, and provide an explanation for observations of sequential recruitment of individual capillaries in response to modulation of terminal arteriolar diameter.

## Introduction

Proper distribution of blood flow within organs is essential for the matching of oxygen and nutrient supply to tissue demand. In the microcirculation, local regulation of blood flow is achieved by contraction and relaxation of the vascular smooth muscle (VSM) surrounding the arterioles and small arteries. Changes in VSM tone are elicited in response to changes in several factors, including levels of oxygen and other metabolites, intraluminal pressure, and luminal wall shear stress (Duling et al. 1987). These vasoactive stimuli not only act locally but also induce responses that are propagated upstream, causing vessels feeding the site of the stimulus to constrict or dilate. The resulting coordinated control of VSM tone in vessels proximal to the affected site contributes to blood flow regulation in response to changes in the metabolic demand of the tissue (Segal et al. 1989; Berg et al. 1997; Cohen et al. 2000).

Blood flow rate is regulated over a particularly wide range in skeletal muscle, which experiences large variations in metabolic demand (Saltin et al. 1998; Segal 2005). How perfusion is matched to demand in skeletal muscle has not been fully resolved. Starting with the work of Krogh (1919a,b), it has been proposed that one of the contributing mechanisms is capillary recruitment, whereby vessels that are not flowing at rest commence flowing in response to increased metabolic demand (Klitzman et al. 1982; Berg et al. 1997). The mechanisms of recruitment, however, remain incompletely understood, and even the occurrence of recruitment is subject to debate (Clark et al. 2008).

Early investigators considered that capillary recruitment resulted from control of flow in individual capillaries by “pre-capillary sphincters” (Krogh 1919a,b). This concept has persisted in textbooks. However, precapillary sphincters are not observed in skeletal muscle (Gorczynski et al. 1978; Lindbom and Arfors 1985; Johnson 1995; Segal 2005). Instead, active control of flow in capillaries of skeletal muscle has been observed to occur only in vessels down to the level of terminal arterioles (Delashaw and Duling 1988; Berg 1995). These observations led to the concept that the smallest unit for control of blood flow in skeletal muscle is the microvascular unit, consisting of a terminal arteriole and group of capillaries fed by it (numbering about 20) (Delashaw and Duling 1988; Emerson and Segal 1997; Lo et al. 2003).

According to this concept, recruitment would be expected to occur at the level of microvascular units, not individual capillaries. However, Lindbom and Arfors (1985) observed a sequential increase in the number of RBC-perfused capillaries fed by a single terminal arteriole during vasodilation elicited by a decrease in ambient oxygen levels. Their observations imply that recruitment of individual capillaries can occur in response to dilation of terminal arterioles. Lindbom and Arfors (1985) attributed this behavior to the existence of a yield shear stress in blood. However, the yield shear stress refers to bulk blood flow and does not apply to red blood cells (RBCs) flowing in narrow capillaries. An alternative potential explanation is that nonlinear rheological effects associated with RBC motion in nonuniform capillaries cause flow cessation. A previous quantitative analysis (Secomb and Hsu 1995) showed that such behavior would occur only at flow velocities much lower than those seen in experiments (Lindbom and Arfors 1985), and cannot account for the observed recruitment. Thus, the mechanism of recruitment at the level of individual capillaries in response to modulation of arteriolar diameters has remained unclear.

The regulation of blood flow in the microcirculation involves a number of interacting physical and biological processes, occurring in a network with a complex and heterogeneous structure. Due to technical limitations, it is not possible to measure many of the relevant variables in individual microvessels. Therefore, theoretical models have played an important role in the investigation of microcirculatory phenomena (Secomb et al. 2008). Such models provide a framework for integrating available information and quantitatively testing hypotheses regarding microcirculatory function. The effects of varying the number and location of perfused capillaries on tissue oxygenation in skeletal muscle have been studied using oxygen transport simulations (Lo et al. 2003; Goldman et al. 2004). These models did not address the mechanisms determining the distributions of flowing vessels. Previous theoretical models of flow regulation (Ursino et al. 1998; Cornelissen et al. 2002; Arciero et al. 2008; Carlson et al. 2008; Kleinstreuer et al. 2008) have generally considered vascular networks as sets of compartments connected in series, where each compartment contains multiple identical vessels of a given type, connected in parallel. Such models do not lend themselves to studying recruitment, which necessarily involves differences in behavior among capillaries, with some ceasing to flow while others continue flowing.

In microvascular networks with heterogeneous structures, experiments have demonstrated unequal partition of discharge hematocrit (H_D_) at diverging bifurcations, such that the daughter vessel with the larger flow rate tends to receive a larger hematocrit than the other daughter vessel (Schmid-Schonbein et al. 1980). This phenomenon, also referred to as phase separation, creates a nonuniform distribution of hematocrit throughout the network. A recent study (Roy et al. 2012) examined metabolic flow regulation in a heterogeneous network derived from the rat mesentery, assuming that the metabolic signal was derived from RBCs. Including phase separation in the model led to different behavior than was predicted if uniform hematocrit was assumed. This shows the importance of considering effects of phase separation when analyzing flow regulation in heterogeneous networks.

The objective of this study is to test the hypothesis that capillary recruitment occurs in heterogeneous microvascular networks as a consequence of local blood flow regulation by changes in arteriolar VSM tone, in combination with unequal hematocrit partition at diverging microvessel bifurcations. This concept was proposed previously (Honig et al. 1980; Schmid-Schonbein and Murfee 2008), but it was not demonstrated that this mechanism provides a quantitative explanation for the observed capillary recruitment. Here, theoretical models are used to simulate blood flow, oxygen transport, and flow regulation in a heterogeneous microvascular network structure that is based on previously published experimental results in the hamster cremaster muscle (Berg 1995). The simulation of blood flow includes effects of phase separation. Oxygen transport is simulated using a method that takes into account the effects of all surrounding vessels on the oxygen level at each point in the tissue (Secomb et al. 2004). The theoretical model for metabolic flow regulation is based on modulation of arteriolar diameters according to the length-tension characteristics of VSM (Carlson and Secomb 2005; Arciero et al. 2008; Carlson et al. 2008; Roy et al. 2012). Responses of VSM tone to myogenic, shear-dependent, and metabolic stimuli are included.

## Methods

### Network structure

**Figure 1:**
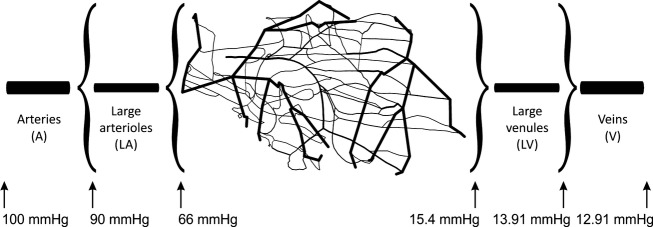
Schematic of the network model. A complete flow pathway through the circulation is formed by an experimentally observed microvascular network, with the addition of upstream and downstream segments representing arteries, large arterioles, large venules, and veins. The distribution of intravascular pressures is shown for the reference state of the network model.

Most previous models of blood flow regulation have described the microcirculation in terms of a set of representative vessel segments (Ursino et al. 1998; Cornelissen et al. 2002; Arciero et al. 2008; Carlson et al. 2008). In such models, the vasculature is divided into several classes of vessels, for example, artery, large arteriole, small arteriole, capillary, small venule, large venule, and vein. The vessels in a particular class are assumed to be equivalent and arranged in parallel, with the different classes arranged in series. This assumption simplifies computations, but does not take into account structural heterogeneities present in actual microvasculature. In the study of Roy et al. (2012), the heterogeneity of network structure was represented by replacing the small arterioles, capillaries, and small venules in the representative segment model with a realistic network structure derived from experimental observations in the rat mesentery. A similar approach is used in this study, as shown in Figure 1. Here, the network structure is derived from observations of the hamster cremaster muscle (Berg 1995; Berg et al. 1997), in which the microvasculature was imaged and mapped in a control state and in a pharmacologically induced maximally dilated state, and RBC fluxes were measured in the arterioles and capillaries. Some vessels had no observable RBC flux in the control state, but observable RBC flux in the maximally dilated state, indicating the occurrence of recruitment. All the vessels with observable RBC fluxes in the control state or the dilated state or both were included in the network maps for the two states (Berg 1995).

From the experimentally obtained maps of network structure in the two states, a single network structure was derived that contained all vessels included in either of the observed maps. This network, which contains 125 vessels, is used as the basis for the present model. The observed maps included arterioles and capillaries up to the points at which they converged to form venules, but did not include information about the location of the venules. In order to complete the network structure, 12 venules were inserted, so as to connect all disconnected capillaries, while minimizing the length of venules added. This procedure resulted in a 137-segment microvessel network. The pressure drop in the venules is typically a small fraction of the overall pressure drop in the network. Moreover, the venules make a minor contribution to the overall oxygen exchange in the network. Therefore, the distributions of blood flow and oxygen transport in the network are relatively insensitive to the assumed geometry of this added venular network.

**Figure 2:**
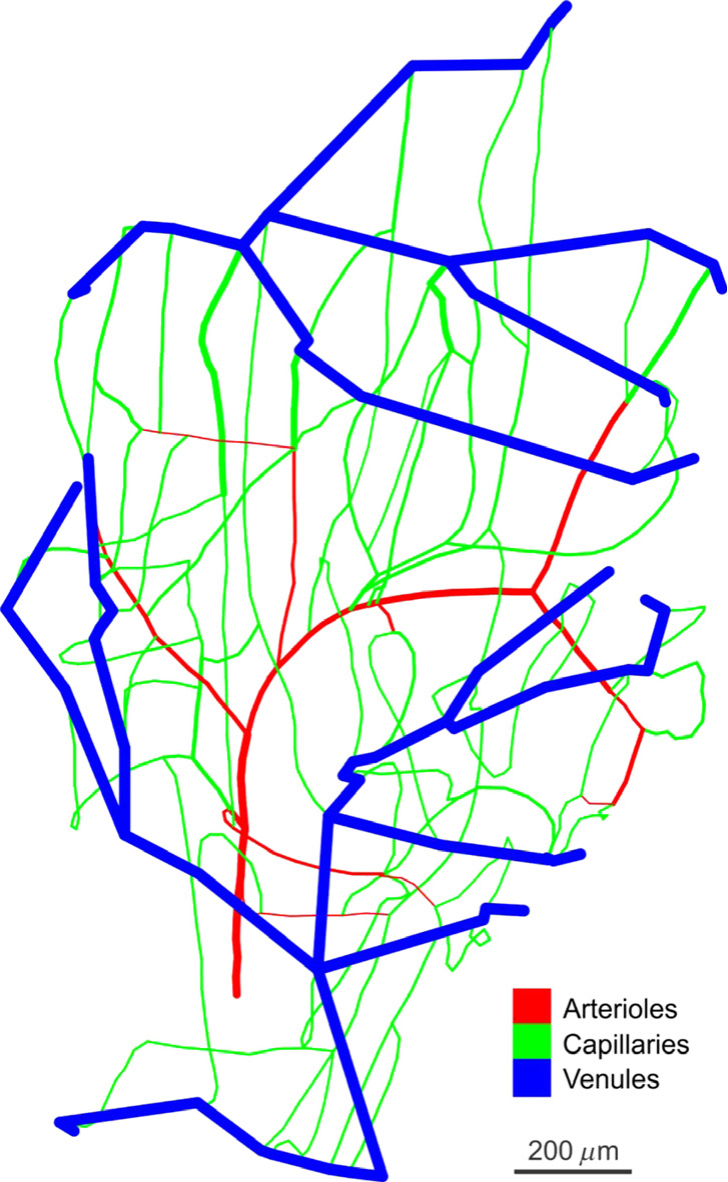
Network used in simulations. Arterioles (red) and capillaries (green) are derived from observations of hamster cremaster muscle (Berg 1995). Venules (blue) are added to form connections with downstream ends of capillaries.

Vessels in the 137-segment microvessel network are classified as arterioles, capillaries, or venules. Arterioles, which are capable of active regulation, are defined based on the branching pattern: If a vessel is the parent vessel at a diverging bifurcation and has a diameter in the dilated state of at least 8 μm, it is classified as an arteriole (Wiedeman et al. 1981). All other vessels (except the added venules) are classified as capillaries. This results in 32 arterioles, 93 capillaries, and 12 venules (Fig. 2). Of the arterioles, six were terminal arterioles, giving about 15 capillaries per terminal arteriole. This is similar to the number of capillaries previously stated to form a microvascular unit (Delashaw and Duling 1988). To form a complete flow pathway through the systemic circulation, two upstream vessels (artery A, and large arteriole LA) and four downstream vessels (large venules LV1, LV2, and veins V1, V2) are added, one of which (LA) is capable of active regulation, bringing the total number of vessels in the simulated network to 143.

### Network hemodynamics

The microvessel network is represented as a set of interconnected segments, each with a defined diameter and length. Pressure-driven flow in each segment and conservation of mass at each node are assumed. Phase separation of RBCs at diverging bifurcations is implemented based on previously derived empirical relationships (Pries et al. 1996). Flow in each segment is governed by Poiseuille's law, with an apparent viscosity that depends on vessel diameter and includes effects of an endothelial surface layer (Pries and Secomb 2008). Using an iterative technique (Young 1954), the resulting nonlinear system of equations is solved at each time step with fixed diameters to obtain the apparent viscosity, flow rate, hematocrit, and wall shear stress in each segment.

In the experimental observations (Berg 1995), vessels were considered to be flowing if they had observable RBC fluxes. As RBC flux was reported in steps of 25 cells/sec, the threshold for observable RBC flux is assumed to be *F*_threshold_ = 12.5 cells/sec. In the following, vessels with RBC flux above this threshold are referred to as flowing and others as nonflowing.

### Flow regulation model

The model for flow regulation is based on the modulation of arteriolar diameters according to the passive and active length-tension characteristics of VSM, and includes myogenic, shear-dependent, and metabolic responses (Arciero et al. 2008; Carlson et al. 2008). Steady-state tension in the vessel wall (*T*_total_) is represented as the sum of a passive component and an active component generated by the VSM (Carlson and Secomb 2005):


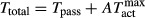
(1)

Here, *A* represents the local activation level of the muscle in each vessel, and has a range from 0 to 1, where *A* = 0 represents no vascular tone and *A* = 1 represents maximal vasoconstriction. The passive tension in the wall of an arteriole with diameter *D* is given by


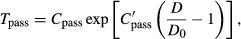
(2)

where *C*_pass_ and *C*′_pass_ are constants representing the magnitude and diameter dependence of passive tension, and *D*_0_ is the passive diameter at an intraluminal pressure of 100 mmHg. The maximal active wall tension is described by


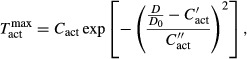
(3)

where *C*_act_, *C*′_act_, and *C*″_act_ are constants representing maximally active VSM peak tension, length dependence, and tension range, respectively. A target activation level is introduced, which is assumed to be a saturating function of the total vasoactive signal, *S*_tone_:


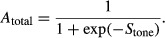
(4)

The parameter *S*_tone_ represents the combined effects of the myogenic, shear-dependent, and metabolic input stimuli (Arciero et al. 2008; Carlson et al. 2008):


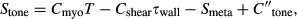
(5)

where *C*_myo_, *C*_shear_, and *C*″_tone_ are constants representing the sensitivity of *S*_tone_ to the various stimuli. The wall tension is given by *T* = *PD*/2, where *P* is the average segment intraluminal pressure, *τ*_wall_ is the wall shear stress, and *S*_meta_ is the conducted metabolic response signal, computed as described below. The model defined by equations (1)–(5) was shown to yield steady-state pressure–diameter relationships in close agreement with experimental observations of myogenic responses in arterioles of various tissues (Carlson and Secomb 2005; Carlson et al. 2008).

**Figure 3:**
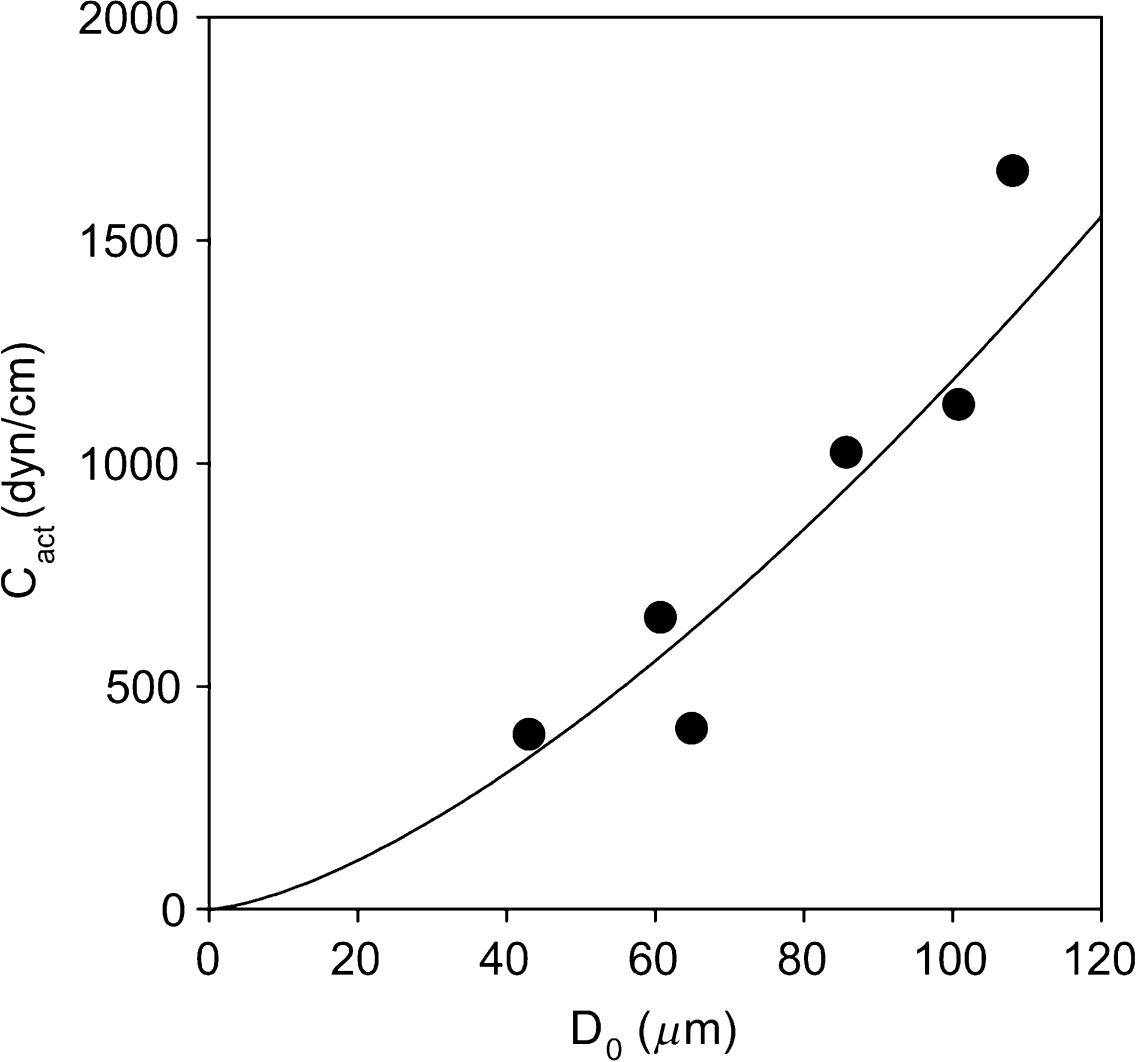
Power law fit of *C*_act_ versus *D*_0_ for six experimental data sets (Carlson and Secomb 2005). Solid circles: experimental data. Curve: power law fit: *C*_act_ = 1.30 *D*_0_^1.48^. In the fitting procedure, the data points were weighted by the number of vessels in each data set.

Parameter values in the model for VSM tone were chosen to match those used previously (Berg 1995; Arciero et al. 2008; Carlson et al. 2008; Roy et al. 2012). For the parameter *C*_act_, the maximally active VSM peak tension, values were needed for smaller diameter vessels (<30 μm in the maximally dilated state) than those considered in the previous models. Therefore, *C*_act_ was estimated by fitting a power law relationship to six data sets (Carlson and Secomb 2005) with the smallest average value of *D*_0_, including four data sets for hamster microvessels (Fig. 3). Values of parameters used in the model are given in Table 1.

**Table 1:** Parameter values used in the model

Description	Parameter	Value	Unit	Reference
Oxygen demand	*M*_0_	0.5 to 8	cm^3^ O_2_/100 cm^3^/min	
Hill equation exponent	*n*	2.55		Klitzman et al. (1988)
Half-maximal Hb saturation	*P*_50_	26	mmHg	Klitzman et al. (1988)
Time constant for diameter	*τ*_d_	1	sec	Arciero et al. (2008)
Time constant for activation	*τ*_a_	20	sec	Roy et al. (2012)
VSM activation tension sensitivity	*C*_myo_	1.369/D_0_	cm/dyn	Carlson and Secomb (2005)
VSM activation shear stress sensitivity	*C*_shear_	0.0258	cm^2^/dyn	Carlson et al. (2008)
VSM activation conducted response sensitivity	*C*_meta_	5000	(1/μM/cm)	Roy et al. (2012)
VSM constant	*C*′′_tone_	−0.908 to 132.1		Carlson and Secomb (2005), Carlson et al. (2008), Roy et al. (2012)
Passive tension strength	*C*_pass_	6.666·*D*_0_	dyn/cm	Carlson and Secomb (2005)
Passive tension sensitivity	*C*′_pass_	−0.027·*D*_0_ + 12.52		Carlson et al. (2008)
Maximally active VSM peak tension	*C*_act_	1.30·*D*_0_^1.48^	dyn/cm	Carlson and Secomb (2005)
Maximally active VSM length dependence	*C*′_act_	−0.00059·*D*_0_ + 0.773		Carlson et al. (2008)
Maximally active VSM tension range	*C*″_act_	−0.00080·*D*_0_ + 0.415		Carlson et al. (2008)
Passive reference vessel diameter	*D*_0_	8 to 20	μm	Berg (1995)
Threshold for observable RBC flux	*F*_threshold_	12.5	cells/sec	Berg (1995)
*P*O_2_ where metabolic signal is half-maximal	*P*_0_	1	mmHg	Secomb et al. (2004)

### Oxygen transport

Previous models for metabolic flow regulation considered oxygen exchange between each vessel and an associated local tissue region (or “tissue sleeve”) of fixed width surrounding the vessel (Arciero et al. 2008; Roy et al. 2012). A limitation of this type of oxygen transport model is that as blood flow rate approaches zero in an individual vessel, the partial pressure of oxygen (*P*O_2_) must go to zero in that vessel. In reality, tissue cells may receive oxygen from any vessel that is sufficiently close. A decrease in flow in a particular vessel does not necessarily cause hypoxia if other well-oxygenated vessels are nearby. Therefore, models based on a fixed oxygen consumption rate per vessel length overestimate the flow-dependent changes in intravascular oxygen levels and exaggerate the ability of metabolic responses to maintain flow in all vessels. Such models are therefore not suitable for investigating vascular recruitment.

Here, a more realistic oxygen transport model is used. The model is capable of simulating a spatially varying oxygen field, including effects of diffusive interaction between all vessels in the network and the entire tissue region. The governing equation in the tissue is as follows:


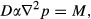
(6)

where *D* and *α* are the diffusivity and solubility of oxygen and tissue, *p* is the *P*O_2_ and *M* is the oxygen consumption rate, which is assumed to follow Michaelis–Menten kinetics:


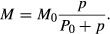
(7)

A Michaelis constant (*P*_0_) of 1 mmHg is used (Secomb et al. 2004). Oxygen demand (*M*_0_) is varied between 0.5 and 8 cm^3^ O_2_/100 cm^3^/min, corresponding to a range of metabolic conditions. Convective oxygen transport in vessels is simulated, including the effects of variable hematocrit and the nonlinear oxygen binding characteristics of hemoglobin. Equation (6) is solved by a Green's function method (Secomb et al. 2004), in which each vessel is represented as a set of discrete oxygen sources and the tissue is represented a set of discrete oxygen sinks. The oxygen field in the tissue is represented as a superposition of the fields resulting from the sources and sinks. This allows for more efficient computation, relative to other computational approaches, as it reduces the number of unknowns needed to represent the oxygen field.

The geometric locations of the added upstream and downstream vessels are not specified. For the purpose of modeling oxygen transport, it is assumed that the arterial vessels lying outside the region with observed network geometry (A and LA) are surrounded by a tissue sleeve of width 18.8 μm in which oxygen is consumed at a fixed rate. The sleeve width is chosen to correspond to a typical measured value of skeletal muscle capillary density of 500/mm^2^ (Arciero et al. 2008). Oxygen exchange by the added venous vessels (LV1, LV2, V1, and V2) is neglected. The Hill equation is used to calculate oxyhemoglobin saturation as a function of vessel *P*O_2_, with parameters of *n* = 2.55 and *P*_50_ = 26 mmHg, based on estimates in hamster cremaster muscle (Klitzman et al. 1983). Oxygen content of all inflowing vessels to the networks is prescribed in terms of the *P*O_2_. At the arterial inflow to the network, a *P*O_2_ of 100 mmHg is assumed. The model includes oxygen loss from arterioles (Duling and Berne 1970). At venular inflows to the network, a *P*O_2_ of 20 mmHg is assumed based on estimates of venular saturation (Ellsworth et al. 1988).

### Conducted metabolic signal

The origins of the metabolic signals involved in local flow regulation are not definitely established. Roy et al. (2012) analyzed flow regulation in heterogeneous microvascular networks, assuming either wall-derived or RBC-derived metabolic signals, and showed that a wall-derived signal was more effective in matching perfusion to local oxygen demand. In reality, several mechanisms likely contribute to metabolic flow regulation, involving wall-derived, tissue-derived, and/or RBC-derived signals. The involvement of mechanisms independent of RBCs has been shown (Ngo et al. 2010). In this study, the metabolic signals in each segment are assumed to be generated by signals from the vessel walls, as this type of signaling was found to be effective for local flow regulation in heterogeneous networks (Roy et al. 2012). Although several potential mechanisms leading to the generation of wall-derived signals are known, their characteristics remain incompletely understood, including their ranges of *P*O_2_ sensitivity and their individual contributions to metabolic regulation under various conditions. Here, it is assumed that the local signal *S*_*loc*_ reflects the local oxygen deficit and is proportional to the difference between oxygen demand (*M*_0_) and oxygen consumption (*M*, given by eq. 7), that is,


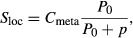
(8)

where *C*_meta_ is a parameter describing the strength of the signal and *P*_0_ = 1 mmHg, corresponding to the Michaelis constant for tissue oxygen consumption.

The signal is assumed to be conducted upstream to the arterioles, resulting in metabolic control of arteriolar tone (Segal et al. 1989), with exponential decay according to a characteristic length constant. Effects of varying the length constant were tested in the model and shown to have little effect, as long as it was not greatly decreased. At bifurcations diverging in the upstream direction, the conducted signal is partitioned in proportion to the vessel diameters; at upstream converging bifurcations, the signal is summed (Roy et al. 2012). The total metabolic signal in each vessel, *S*_meta_, is the sum of the propagated signal from downstream vessels and the signal generated locally (*S*_loc_). Virtual segments are added distal to draining vessels to generate conducted signals at the network outflows.

### Dynamics of arterioles

The dynamic behavior of each arteriole is described by differential equations for the variation with time of diameter and activation (Arciero et al. 2008; Carlson et al. 2008; Roy et al. 2012):


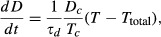
(9)


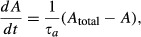
(10)

where *D*_*c*_ and *T*_*c*_ are the diameter and wall tension in the reference state (described below), and *τ*_d_ and *τ*_a_ are time constants. In these simulations, the eventual behavior of the system is analyzed, not the transient response, so the assumed values of the time constants do not generally affect the results. Available data indicate that *τ*_d_ is around 1 sec, and *τ*_a_ is between 1 sec and 1 min, so an intermediate value (20 sec) was used (Roy et al. 2012). Equations (9) and (10) are integrated from *t* = 0 to 200 sec using an explicit Euler method. After 200 sec, the system is found either to reach a steady state or to show stable oscillations, consistent with occurrence of vasomotion (Arciero and Secomb 2012). For purposes of analysis, the final values of system variables are defined by averaging over the interval from 100 to 200 sec. The simulation of oxygen transport is the most computationally demanding part of the calculation. The iterative method used in the Green's function method at each time step involves solutions of large linear systems, which can be implemented with parallel algorithms. Simulation of 200 sec of real time requires 800 computational time steps, with each time step taking 10 to 60 sec of computer time using a graphical processing unit–based parallel processing system.

### Reference state

A reference state is defined for the network with a moderate level of arteriolar tone, corresponding to a relatively low level of oxygen consumption in the skeletal muscle. The distribution of pressure drops in the network model is chosen to correspond where possible to the previous model (Arciero et al. 2008), with pressure drops in the A, LV, and V segments of 10, 1.49, and 1 mmHg. The pressure drop across the microvessel network is chosen to minimize the total squared variation between vessel RBC fluxes in the reference state and those observed experimentally data in the control state (Berg 1995), resulting in a pressure of 66 mmHg at the arterial side of the microvessel network. This was achieved by adjusting the length of the LA segment. The arterial inflow and venous outflow pressures are set to 100 and 12.91 mmHg, respectively (Roy et al. 2012). The resulting distribution of pressures is indicated in Figure 1.

To establish the distribution of tone in the reference state, values of *S*_tone_ in each arteriole are determined as follows. If an arteriole had observable RBC flux in the maximally dilated experimental network (Berg 1995), then the passive vessel diameter at a pressure of 100 mmHg (*D*_0_) is set as the measured diameter in the maximally dilated state. If an arteriole had observable RBC flux also in the control experimental network (Berg 1995), then the diameter in the model reference state (*D*_*c*_) is set as the measured diameter in the control state. The reference state is assumed to be at equilibrium, so that *T* = *T*_total_ and *A* = *A*_total_, which allows for an explicit calculation of *S*_tone_ in each arteriole based on equations (1) and (8):


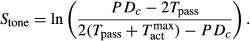
(11)

For some vessels in the experimental data set, the diameter in the maximally dilated state was less than or equal to the diameter in the control state. Such behavior might result from a decrease in pressure in some arterioles due to overall vasodilation. In these cases, it is assumed that the vessels are close to maximal dilation in the reference state; that is, *D*_c_ ≍ *D*_0_, and *S*_tone_ is set to a large negative value (−1000) so that *A* ≍ 0. Some arterioles had observable RBC fluxes only in the dilated state of the network. In the reference state, these arterioles are assigned small diameters *D*_*c*_ in the range 2.9–3.8 μm, such that all have RBC fluxes below *F*_threshold_.

Of the capillaries, 32 were observed as flowing only in the dilated state, implying that they should have RBC fluxes less than *F*_threshold_ in the reference state, but greater than *F*_threshold_ when arteriolar diameters are set to their dilated values. When arteriolar diameters were initially set to their *D*_*c*_ values as described above, only 11 of the 32 capillaries had RBC fluxes less than *F*_threshold_ in the simulated reference state. The absolute root mean square (RMS) error in individual diameter measurements in microvessel networks was estimated as ±1.2 μm based on imaging limitations (Pries et al. 1994). Therefore, small adjustments (≤1.2-μm increase or decrease) in capillary diameter were made where this led to an increase in the number of nonflowing capillaries in the reference state. After these changes were made, 19 of the 32 capillaries flowing only in the dilated state are nonflowing in the reference state, and all 32 are flowing when arteriolar diameters are set to their dilated values. In total, of the 125 capillaries and arterioles in the microvessel network, 27 (21.6%) are nonflowing in the reference state, and therefore have the capacity for recruitment.

As is evident from the above description, the procedure for defining the control state involves a number of assumptions. The resulting reference state may not accurately represent, at the level of individual vessels, the conditions that existed in the experimental preparation. However, the procedure used was designed to ensure that distributions of geometric and hemodynamic parameters and their degree of heterogeneity in the reference configuration are similar to those existing in the in vivo preparation. The purpose of the reference state is to provide an internally consistent network with realistic properties as a reference configuration, which serves as a control for further simulations in which hemodynamic or metabolic parameters are varied.

## Results

**Figure 4:**
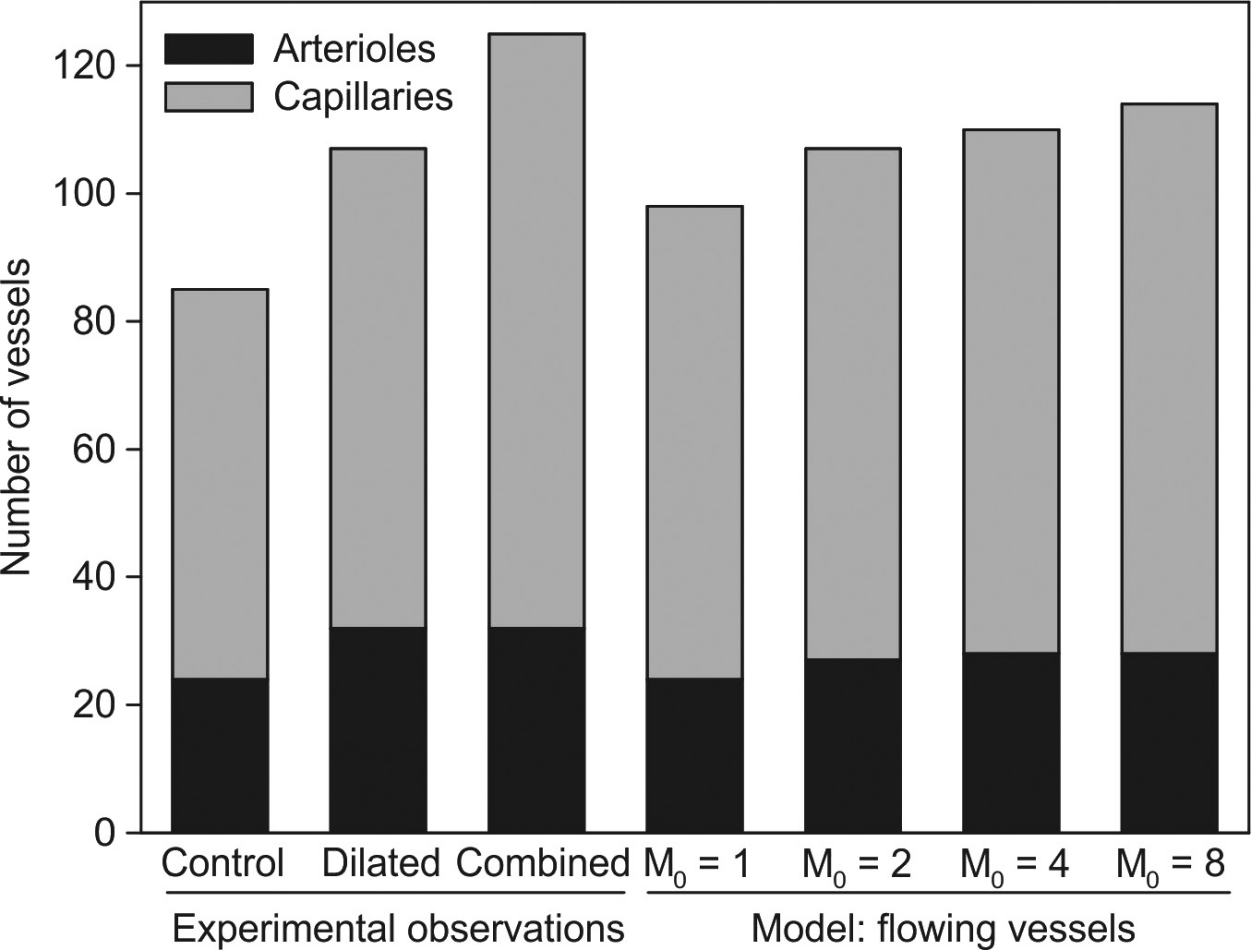
Numbers of vessels in experimentally observed networks and numbers of flowing vessels in model simulations.

The numbers of flowing arterioles and capillaries observed experimentally and predicted by the model are shown in Figure 4. In the experimental observations, the total number of flowing vessels increased by 26% (from 85 to 107) between the control and vasodilated states. The number of flowing arterioles and capillaries in the dilated state (107) is fewer than the total number of such vessels in the combined network consisting of the union of the networks in the control and vasodilated states (125) because a number of vessels (18) were observed to be flowing in the control state but not in the dilated state. Such behavior may result from hemodynamic effects including redistribution of hematocrit with vasodilation, or it may reflect incomplete visualization of all flowing vessels in the network in the experiments. The combined network is used as the basis for the model simulations.

**Figure 5:**
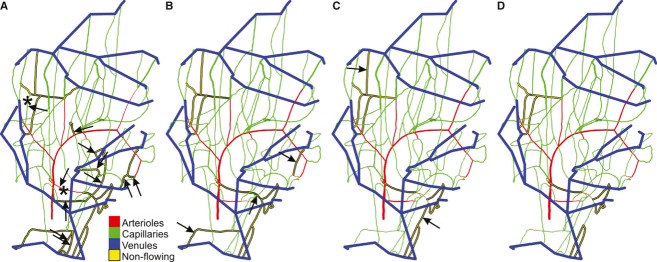
Spatial distributions of nonflowing vessels and flowing arterioles, capillaries, and venules, when (A) *M*_0_ = 1; (B) *M*_0_ = 2; (C) *M*_0_ = 4; (D) *M*_0_ = 8 cm^3^ O_2_/100 cm^3^/min. Black arrows indicate vessels that start flow with an increase in oxygen demand. Black arrows with an asterisk in panel A indicate vessels that stop flow with an increase in oxygen demand.

As shown in Figure 4, the model predicts that the numbers of flowing arterioles and capillaries increase progressively with increasing oxygen demand. This supports the hypothesis that vessel recruitment can occur as a consequence of local metabolic regulation of blood flow in a network with heterogeneous structure. Overall, the number of flowing arterioles and capillaries increases by about 16% (from 98 to 114), as demand (*M*_0_) is raised from 1 to 8 cm^3^ O_2_/100 cm^3^/min. The spatial distributions of flowing and nonflowing vessels are shown in Figure 5 for oxygen demand *M*_0_ of 1, 2, 4, and 8 cm^3^ O_2_/100 cm^3^/min. In general, increasing oxygen demand causes transitions from nonflowing to flowing states, but a few vessels stop flow with increasing demand (Fig. 5A). In many instances, recruitment or derecruitment of individual capillaries, rather than groups fed by a single terminal arteriole, is predicted with changes in oxygen demand.

**Figure 6:**
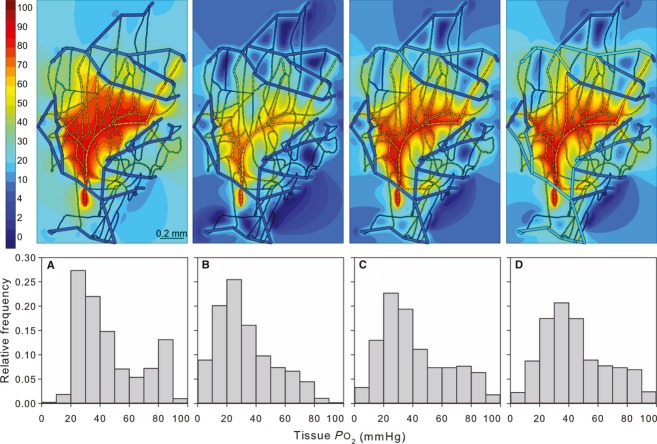
Contour plots (top panels) and histograms (bottom panels) of tissue *P*O_2_ distribution at hundreds of tissue points. Oxygen levels in the vessels and in the surrounding tissue are color-coded according to the scale at left (in mmHg). (A) *M*_0_ = 1 cm^3^ O_2_/100 cm^3^/min. (B) *M*_0_ = 4 cm^3^ O_2_/100 cm^3^/min, with constant hematocrit and without metabolic flow regulation. (C) *M*_0_ = 4 cm^3^ O_2_/100 cm^3^/min, with constant hematocrit and with metabolic flow regulation. (D) *M*_0_ = 4 cm^3^ O_2_/100 cm^3^/min, with nonuniform hematocrit and with metabolic flow regulation.

The effects on predicted tissue oxygenation of including flow regulation and hematocrit partition in the model are illustrated in Figure 6, as spatial distributions (upper panels) and frequency distributions (lower panels). For the reference state, with oxygen demand *M*_0_ = 1 cm^3^O_2_/100 cm^3^/min, virtually no hypoxic tissue is predicted (Fig. 6A). When oxygen demand is increased to *M*_0_ = 4 cm^3^ O_2_/100 cm^3^/min, without metabolic flow regulation (i.e., *S*_meta_ = 0 in each vessel) or phase separation included in the model, the distribution of tissue *P*O_2_ is shifted toward lower levels (Fig. 6B). When metabolic flow regulation is included (Fig. 6C), the tissue *P*O_2_ distribution shifts toward higher levels compared to the case without metabolic flow regulation. Figure 6D shows the distribution with both metabolic flow regulation and phase separation included. The inclusion of nonuniform hematocrit distribution further shifts the tissue *P*O_2_ distribution to higher levels.

**Figure 7:**
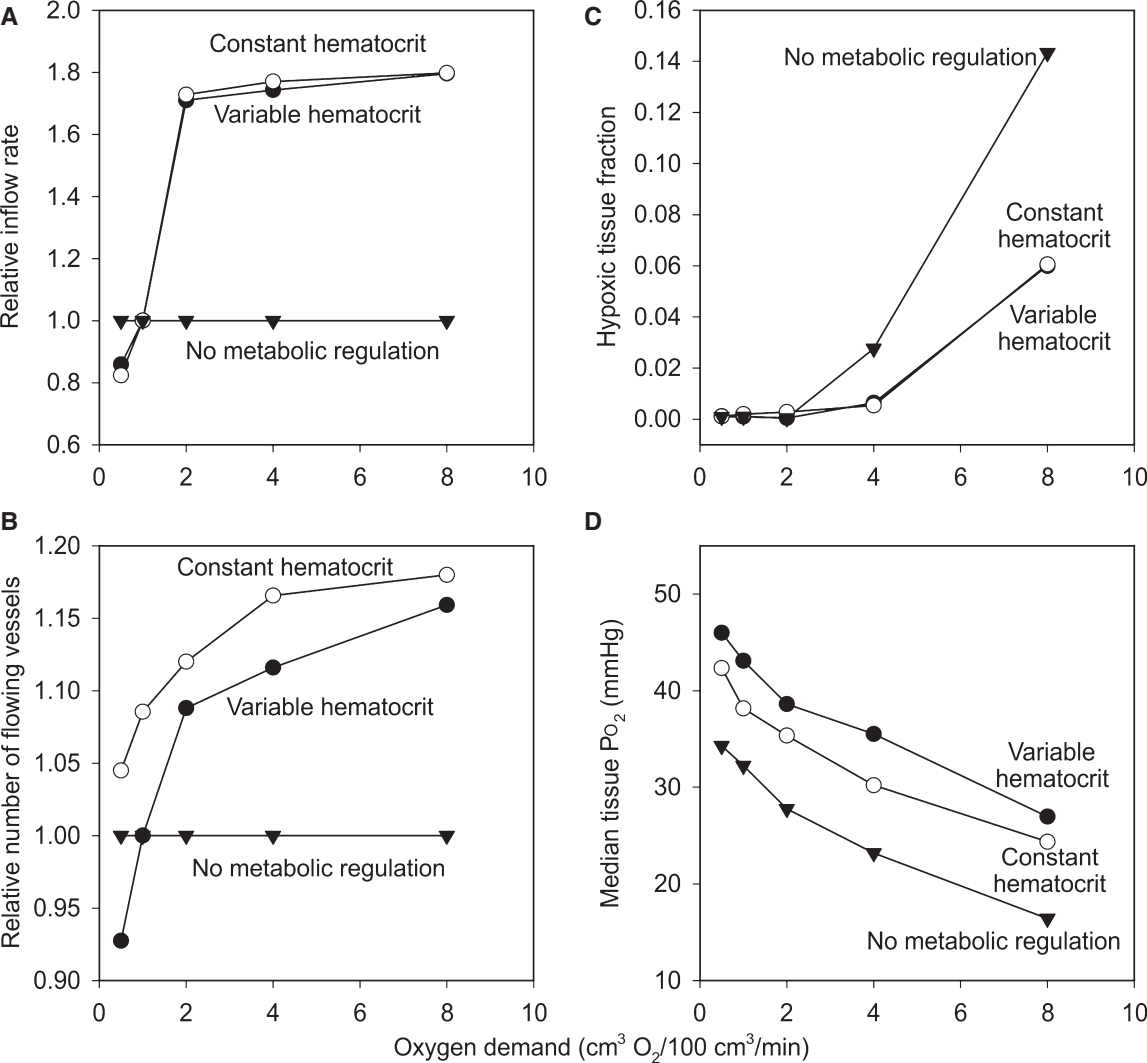
Variables describing network blood flow and tissue oxygenation, as functions of oxygen demand. (A) Relative arteriolar inflow rate. (B) Relative number of flowing vessels. (C) Hypoxic tissue fraction (defined as *P*O_2_ < 1 mmHg). (D) Median tissue *P*O_2_. (•) Variable H_D_ (with phase separation) with metabolic flow regulation. (○) Constant H_D_ (no phase separation) with metabolic flow regulation. (▼) Constant H_D_ without metabolic flow regulation.

In Figure 7, variables reflecting blood flow and tissue oxygenation are plotted as functions of oxygen demand from *M*_0_ = 0.5 to 8 cm^3^ O_2_/100 cm^3^/min for three cases: constant hematocrit without metabolic regulation, constant hematocrit with metabolic regulation, and variable hematocrit with metabolic regulation. Figure 7A shows total blood flow to the network. With metabolic regulation, inflow rate increases as oxygen demand is increased. Introducing phase separation has little effect on total flow. Figure 7B shows the number of flowing arterioles and capillaries. The number of vessels with RBC flux above *F*_threshold_ is lower when phase separation is included. The number increases 13% between the lowest and highest oxygen demand states with constant hematocrit, whereas the increase is 25% with phase separation. Thus, phase separation increases the extent of vessel recruitment as oxygen demand is increased. As expected, the network inflow rate and the number of flowing vessels are independent of oxygen demand when metabolic flow regulation is not included (Fig. 7A and B).

Median tissue *P*O_2_ declines with increasing oxygen demand (Fig. 7D). Inclusion of metabolic flow regulation leads to an increase of about 10 mmHg in median tissue *P*O_2_. The increase is larger when phase separation is included, relative to the uniform hematocrit case. These differences are present for the entire range of oxygen demand considered. Figure 7C shows the hypoxic fraction (fraction of tissue with *P*O_2_ < 1 mmHg). For oxygen demand up to *M*_0_ = 2 cm^3^ O_2_/100 cm^3^/min, there is less than 1% hypoxia in all cases. However, for *M*_0_ = 4, with no metabolic flow regulation, the hypoxic fractions are 0.031 and 0.028 for variable H_D_ (not shown) and constant H_D_, respectively. When metabolic flow regulation is included, the hypoxic fraction is much lower, with values of 0.006 and 0.005, respectively. Similarly, for *M*_0_ = 8, the hypoxic fractions without metabolic flow regulation increase to 0.14 (for both variable and constant H_D_) compared to 0.06 (variable H_D_) and 0.061 (constant H_D_) with metabolic regulation included. Thus, for *M*_0_ > 2, tissue hypoxic fraction is much lower when metabolic flow regulation is included, as would be expected.

## Discussion

Metabolic regulation is necessary for matching blood flow to widely varying oxygen demand in striated muscle. Capillary recruitment, defined as variation in the number of capillaries containing flowing RBCs, is observed to occur with changes in metabolic demands and is considered to contribute to metabolic control of blood flow. This study uses a theoretical model to test the hypothesis that vessel recruitment can occur as a consequence of local changes in arteriolar tone, acting in combination with phase separation at diverging microvessel bifurcations. The results support the hypothesis. When applied to a heterogeneous network structure in striated muscle derived from a hamster cremaster preparation (Berg 1995), the model predicts that metabolic vasodilation not only increases overall flow rate (Fig. 7A) but also increases the number of flowing vessels (Figs. 5, and 7B) defined in terms of RBC flux above a threshold value. With increasing oxygen demand, vessel recruitment is predicted at both the capillary and arteriolar levels.

As discussed in the Introduction, the mechanism of recruitment has been unclear. The most distal site for control of capillary perfusion in skeletal muscle is the terminal arteriole, which controls flow to a microvascular unit comprising multiple capillaries (around 20), whereas recruitment of individual capillaries is observed. This study provides a potential resolution to these apparently conflicting observations. In the simulations, recruitment and derecruitment of individual capillaries are predicted to occur as a consequence of arteriolar dilation and constriction. Here, as in experimental studies, capillary perfusion is defined in terms of RBC flux above a minimum observable threshold. In a heterogeneous network structure, the unequal partition of hematocrit at diverging bifurcations causes vessel hematocrits to vary depending on the flow distribution. As a consequence, recruitment of capillaries within a single microvascular unit can occur sequentially, rather than synchronously, during gradual vasodilation of the feeding arteriole. According to this concept, modulation of flow and vascular recruitment are two aspects of the same physiological response, and it is not meaningful to quantify their individual contributions to the metabolic regulation of flow.

The effects of metabolic flow regulation, with and without unequal hematocrit partition at vessel bifurcations, on predicted hemodynamic and oxygen transport characteristics are illustrated in Figure 7. Including phase separation has little effect on the total flow to the network (Fig. 7A), as variations in individual vessel flow rates tend to cancel when aggregated. However, phase separation has a substantial effect on the number of flowing vessels (Fig. 7B). With phase separation, a larger number of vessels have low hematocrit and low RBC fluxes, and are classified as nonflowing. The extent of recruitment and derecruitment is greater with phase separation because decreases in either flow rate or vessel hematocrit may cause the RBC flux to decrease below the threshold level.

**Figure 8:**
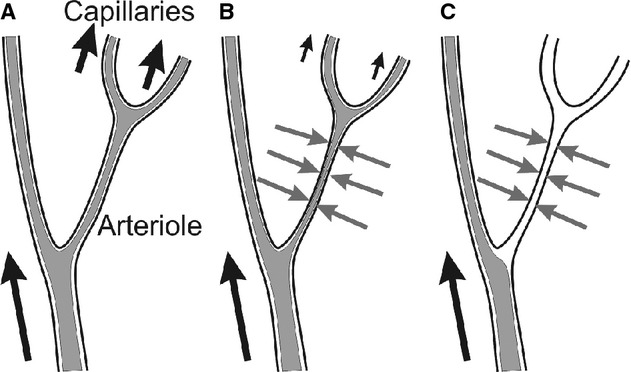
Schematic diagram showing two mechanisms for capillary recruitment and derecruitment in response to changes in arteriolar diameter. Black arrows indicate magnitude of RBC flux. Gray arrows indicate arteriolar vasoconstriction. Gray areas within vessels represent regions containing RBCs. (A) Initial configuration with dilated arteriole. (B) Effect of vasoconstriction without phase separation. Derecruitment may occur because RBC flux drops below threshold level. (C) Effect of vasoconstriction with phase separation. Derecruitment may occur because hematocrit entering the arteriole drops to a low level or zero.

These dual mechanisms contributing to the distribution of RBC flux in microvascular networks and the recruitment and derecruitment of capillaries are illustrated schematically in Figure 8. Regardless of whether phase separation is included in the model, low arteriolar flows may result in capillaries with RBC fluxes below *F*_threshold_ that can be recruited when arteriolar flow increases in response to increased oxygen demand. A second mechanism becomes evident itself only when phase separation is included. Capillary RBC fluxes below *F*_threshold_ may occur because a parent arteriole receives a low hematocrit. With vasodilation, the arteriole may receive a higher hematocrit, leading to recruitment of the capillaries that it feeds. Capillary recruitment is thereby augmented as a result of unequal hematocrit partition at vessel bifurcations.

An unexpected model prediction is that inclusion of phase separation in the model with metabolic flow regulation leads to improved tissue oxygenation, relative to the behavior without phase separation (i.e., with constant hematocrit), as shown by the shift to higher values in the distribution of *P*O_2_ (Fig. 6C and D) and the median tissue *P*O_2_ (Fig. 7D). This improvement in oxygenation occurs despite the fact that fewer vessels are flowing when phase separation is included (Fig. 7B). Phase separation is thus seen to contribute to metabolic flow regulation. By directing increased hematocrit preferentially to vessels with increased flow rates, it provides a mechanism to amplify the changes in RBC flux resulting from modulation of arteriolar diameters. In the present simulations, the inclusion of phase separation has only slight effects on the fraction of hypoxic tissue (Fig. 7C). However, the hypoxic regions are small and lie mainly near the boundaries of the network (see Fig. 6), and so this result may not be representative of the behavior that would occur with more widespread hypoxia.

In the present model, the signal for metabolic regulation in response to changes in oxygen levels is assumed to be generated in the vessel wall, according to equation (8). A previous theoretical study (Roy et al. 2012) examined the distribution of blood flow and tissue oxygenation in a heterogeneous network, assuming that the signal for metabolic flow regulation originated within RBCs. In that case, inclusion in the model of phase separation resulted in poorer oxygenation because a reduction in RBC flux to a given vessel generally caused a reduction in the RBC-derived metabolic signal, thereby exacerbating the reduction in flow. Taken together, these models show that phase separation can have a substantial effect on local metabolic flow regulation, but its effect depends on the metabolic signaling mechanisms involved.

Estimates of the capacity for capillary recruitment in skeletal muscle vary widely (Clark et al. 2008). In some experiments, it has been found to be at least 100% and possibly as high as 200%, relative to the number of flowing capillaries at rest (Honig et al. 1982; Lindbom 1983). In the present simulations, almost 80% of the arterioles and capillaries in the network are flowing in the reference state, and so the capacity for recruitment is limited to about 25%. Furthermore, the range of flow regulation in this model study is restricted according to the observed changes in diameter between the two states of the cremaster preparation (Berg 1995). The physiological range of flow rates in the cremaster muscle is not as wide as that in skeletal muscle (Segal 2005). The geometry of the cremaster muscle, with layers of fibers running in different directions, differs from that of skeletal muscle. Despite these differences, there is no reason to expect that the recruitment mechanism described here would not also be operative in skeletal muscle. The ranges of flow modulation and capillary recruitment possible with the hypothesized mechanisms have no inherent restrictions. Given a network with more vasoconstriction and fewer flowing capillaries in the resting state, the present model would predict a wider range of flow regulation and vascular recruitment.
